# Developmental aspects of maternal-fetal, and infant gut microbiota and implications for long-term health

**DOI:** 10.1186/s40748-015-0007-4

**Published:** 2015-02-11

**Authors:** Josef Neu

**Affiliations:** College of Medicine, University of Florida, 1600 S.W. Archer Road, Gainesville, Florida 32610 USA

**Keywords:** Microbiome, Fetus, Neonate, Development

## Abstract

**Background:**

Early life interactions between the human host and microbes set the stage for future health and disease.

**Findings:**

In this review, some of the relationship of the human microbiome effects will be discussed as they relate to preterm delivery, early life diseases seen in prematurely delivered infants, and other childhood and adult maladies which include autoimmunity, allergic diseases, obesity or a healthy phenotype.

**Conclusion:**

Although the data in these areas is just beginning to emerge, this review will provide a brief summary of some of the key research being done and will also speculate on emerging areas where major questions are being raised.

## Findings

At no stage of a human’s lifetime is the interaction between microbes and the host as precarious as during fetal life, infancy and early childhood where critical windows exist for later life programming, which will dictate the subsequent health of the individual. It is becoming clear that such programming is not solely dependent on the nucleotide base pairing sequence of the human genome, which is composed of only approximately 23,000 genes. Our microbiome, termed by some the “second genome”, comprises a vastly larger number of cells and genes than those derived from the human gametes. Microbes comprising this biome divide rapidly, and have a great potential for environmentally induced alteration. Furthermore, these microbes, most of which are symbionts and commensals are now recognized to exert major immunologic and metabolic effects on the human host.

### The in-utero microbiome

Evidence contradicts the dogma that the fetus resides in a sterile environment and that the newborn only attains its microbiota after exposure to the extrauterine environment [1]. It is been known for over 30 years that even without a ruptured amniotic sac, amniotic fluid frequently contains significant levels of bacteria [2]. Intrauterine “infection” and a subsequent fetal inflammatory response has been linked to prematurity, brain, lung and eye disease after preterm birth [3,4]. Studies in the past decade using non-culture based techniques further reveal a significant presence of microbes in placenta, amniotic fluid and meconium [5-9]. These microbes appear to differ depending on the stage of fetal maturity [5,7]. Since preterm delivery is often associated with an inflammatory response, the finding that greater degree of prematurity is directly related to the bacterial DNA load in amniotic fluid suggests a relationship [6,10]. Causality in this relationship is further supported by the presence of microbes directly correlated with increased levels of white blood cells and interleukin-6 (IL-6) in the amniotic fluid, suggesting a pathophysiologic sequence of increased microbial load, inflammation and preterm birth [10]. A similar study also showed that difficult to culture microbial taxa are present in amniotic fluid and this related to higher levels of IL-6, chorioamnionitis, funisitis and early onset sepsis [9].

Although it has been known that the placenta frequently harbors microbes, and that these microbes are associated with prematurity, recent studies using DNA sequencing to interrogate the placental microbiota from infants born at various gestational ages have been performed [5]. A relationship between different taxa of microbes and level of prematurity as well as infections early in pregnancy were found. Intriguingly a similarity between placental microbes and those found in the mouth from the human microbiota database was discovered. This intriguing finding related placental samples derived from pregnant mothers. However, mouth microbiota samples were derived from a separate non-pregnant female database and not the same pregnant mothers. This requires additional studies wherein samples from different sites in the same woman are analyzed. The fact that *E. coli* was one of the largest taxa represented in the placental samples is of interest in that this is a common resident of the intestine and thus suggests a potential intestinal origin.

It is common dogma that the baby’s first stool (meconium) is sterile. Studies demonstrating that meconium from both preterm and term neonates frequently contain cultivatable and non- cultivatable microbes refute this notion [7,8,11,12]. The origin of these microbes is unknown, but tenable support for the hypothesis that these are derived from swallowed amniotic fluid has been bolstered by recent studies that show a high degree of similarity between meconium microbes in those derived from the literature to be found in amniotic fluid [7]. As with the aforementioned placenta studies, this finding also requires validation using multisite analysis from the same pregnant woman.

**Figure 1:**
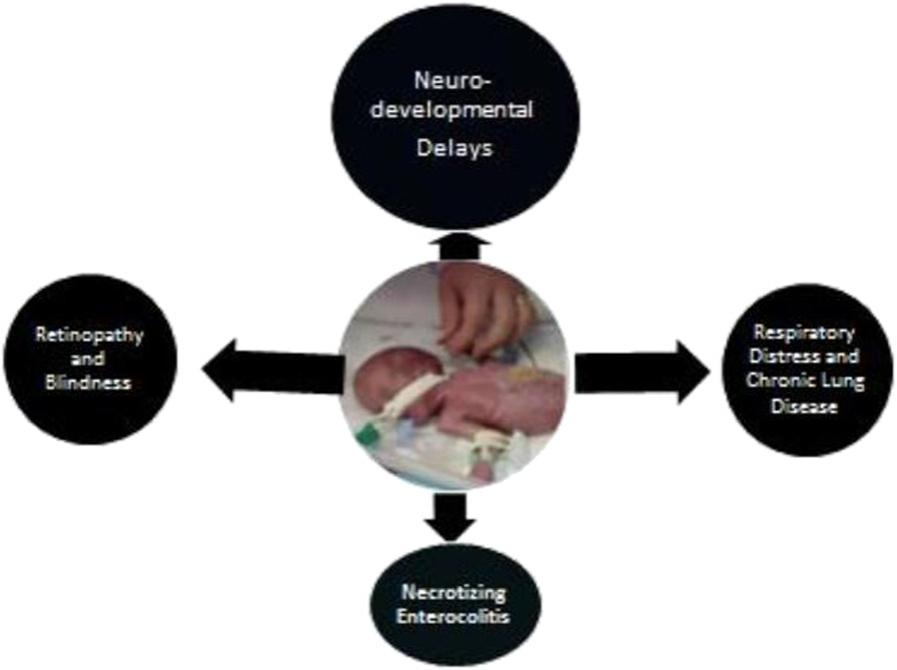
**Morbidities related to preterm birth also relate to prenatal dysbiosis.** The fetal immune system and inflammatory responses are rapidly evolving before birth. The microbial environment may enhance or blunt immune and inflammatory responses.

Thus, the classic dogma that the newborn emerges from a sterile environment should be considered a shortsighted assumption. It is clear that the fetal immune system, especially that of the fetal intestine is rapidly evolving during gestation and that postnatal morbidities such as necrotizing enterocolitis, chronic lung disease, brain white matter disease and other inflammatory conditions are likely to be affected by microbial colonization that occurs well before the infant leaves the uterus (Figure 1).

One concept of in utero colonization has been that microbes residing in the vagina ascend to the chorio-decidual membranes, where they translocate into amniotic fluid [13,14]. One hypothesis suggests that an inflammatory response originating from the decidua leads to preterm labor. Although this remains a commonly held belief, studies suggest that inflammation in decidua is not a cause of preterm labor, but rather is caused by labor [15]. An alternative source of the inflammatory response that has not been given much attention is the fetus. The fetus is exposed to microbes through the fetal skin, lungs, and gastrointestinal tract. Fetal gastrointestinal tract exposure to these microbes appears to be of particular interest because large quantities of amniotic fluid are swallowed during the last stage of pregnancy and thus expose the fetal intestine to these microbes [16,17]. The fact that the fetal intestine is a highly immunoreactive organ [18] that may elaborate an inflammatory response to these microbes, which in turn may ultimately be responsible for triggering preterm labor is intriguing and begs additional studies.

The site of entry of the microbes in the placenta, amniotic fluid and fetus remains unclear. However, a recent study contradicts the notion that the microbes originating from the vagina are the origin of preterm labor [19]. Other potential hematogenous sources are the maternal mouth via inflamed periodontal tissue and the maternal intestinal tract [13]. If, in fact, the maternal intestine is the origin of the microbes that are found in the fetus and in the uterus, and if these indeed are related to inflammatory responses that originate in the fetus that may induce preterm labor, this offers an exciting area for interventions that include maternal dietary manipulation and microbial therapeutic alterations by providing certain microbes into the mother’s diet [20] or perhaps even very specific anti-microbial therapies that prevent those microbes associated with preterm labor from thriving in the maternal intestine and consequently the placenta, amniotic fluid and the fetus.

In summary, pregnancy is the beginning of bacterial exposure for the developing embryo and fetus. The origins of these bacteria include the maternal gastrointestinal tract and these bacteria have been isolated in umbilical cord blood, amniotic fluid placenta, fetal membranes and meconium. These microorganisms in many cases have been associated with no clinical evidence of infection in either the mother or the fetus. One can speculate that the microbiota to which the fetus is exposed may stimulate an inflammatory response, and depending on the types of microbes present, stimulate preterm labor. Furthermore, these bacteria or their components may also contribute to immunologic stimulation that may lead to subsequent injury to the fetus and neonate [4,21] or even immunologic tolerance [22] that leads to prevention of certain diseases such as those associated with autoimmunity and allergy in later life.

### Pre and perinatal factors affecting the developing microbiome

Given that prenatal microbiota have profound effects on the fetus and subsequent development, various mechanisms of how these microbes relate to the host remain an exciting area for research. Since this is such a critical window for development, the interaction of microbes with the host and with environmental perturbations such as maternal diet, antibiotics, and other microbial exposures may play a highly significant role (Table 1).

**Table 1:** **Factors that may affect the newborn microbiome and subsequent development**

**Prenatal**	
	Maternal hygiene
	Maternal Dental Hygiene
	Maternal diet
	Maternal infection
	Maternal antibiotics
**Perinatal**	
	Maternal antibiotics
	Mode of delivery
	Presence of Labor
**Postnatal**	
	Bathing
	Skin to skin mother care
	Incubator
	Caretaker handwashing
	Breastfeeding status
	Heat Treatment of Milk
	Infection
	Mechanical ventilation
	Invasive central lines
	Indwelling feeding tubes

While it may be enlightening to determine the taxonomy of microbes present during this highly vulnerable time, even more important is a better understanding of the function of these microbes and their interaction with the host. The microbial metabolites and immunologic responses in mothers’ gastrointestinal tracts and genitourinary systems and mouth may have profound effects on the fetus. Various metabolites such as short chain fatty acids may be highly epi-genetically active and thus play a major role in determining subsequent phenotypes when the fetus reaches later age.

Studies in both animals and humans are beginning to support these notions. Autoimmune diseases such as type I diabetes may be affected by such maternal environmental factors as type of diet and antibiotic treatment. For example, a study in pregnant non-obese diabetic (NOD) mice fed a gluten-free or standard diet until the pups were weaned to a standard diet showed that the early life gluten-free environment decrease the incidence of diabetes and insulitis in the offspring [23]. This was associated with differences in the intestinal microbiota of the mothers who were fed the gluten-free diet. Of interest is that pancreatic regulatory T cells were increased in the gluten-free fed offspring as were tight junction related genes in the intestine, which also corresponded to decreased intestinal gene expression of pro-inflammatory cytokines. Another study using the NOD diabetic mouse model involved treatment of pregnant mothers with a cocktail of antibiotics and investigation of T lymphocyte populations in the offspring [24]. The offspring from NOD mothers treated with antibiotics during gestation showed a reduction in the diversity of the gut microbiota, as would be expected. In addition, the offspring of mothers treated with antibiotics also showed immunologic alterations of the gut immune system which potentially could become a risk factor for the development of diabetes in these animals.

Cesarean section versus vaginal delivery also appears to affect subsequent microbial colonization in the intestine of the newborn and infant. A study by Dominguez Bello [25] using 16SrRNA sequencing suggested that vaginally delivered infants acquired bacterial communities resembling their own mother’s vaginal microbiota whereas C-section infants were colonized by bacterial communities similar to those found in the mother’s skin. Of interest is that in this study the differences were seen in meconium, the infants first stool, which would more likely represent the in utero environment rather than whether the infant passed through the birth canal or was born by cesarean section. This raises the question of whether the mothers need for a C-section versus vaginal delivery may have been responsible for differences in the microbiota in these infants, rather than the mode of delivery. Nevertheless, a Canadian study evaluated fecal microbiota at four months of age [26] in healthy infants who were born by either C-section versus vaginal delivery. Differences were seen in the fecal microbiota of infants born by C-section versus vaginal delivery at the phylum level at four months after birth. Further evaluation revealed that most of these differences were seen in infants who were fed with formula. Those infants receiving mothers’ milk exhibited only minor differences at four months whether or not they were born by C-section versus vaginal delivery. In another study done at the Karolinska Institute in Sweden [27], major differences in microbial diversity were seen up to two years after birth in the fecal microbiota of infants who had been delivered by cesarean section versus vaginal delivery. There was a preponderance of Bacteroidetes in the infants born by vaginal delivery and this was seen in parallel to higher levels of anti-inflammatory mediators in the same infants who had the higher levels of Bacteroidetes. These microbes are known to have in their cell walls a protein which stimulates the production of these anti-inflammatory mediators [28].

Since several epidemiologic studies suggest a greater preponderance of autoimmune diseases, celiac disease, obesity and other disorders in infants born by cesarean section [29], this raises the question of whether increasing levels of cesarean section may become a public health issue in areas where C-sections are highly prevalent. This is especially important in some countries where the prevalence of cesarean section has been approaching very high levels, up to 80%.

### The preterm neonate

#### Enteral versus parenteral feeding

In neonatal intensive care, there has been a reluctance to utilize the neonatal gastrointestinal tract for feeding infants, largely because of the fear of necrotizing enterocolitis (NEC) and also because many of these infants experience various levels of feeding intolerance. Instead, these infants are nourished with intravenous parenteral nutrition, which does provide nutrients to the rapidly developing preterm infant, but is suboptimal in terms of supporting intestinal mucosal growth and development. At the same time that the infant gastrointestinal tract is not being directly nourished with enteral feedings, microbes residing in the gastrointestinal tract of these infants also are exposed to a lack of nutrients. This raises the question of whether the lack of nutrient substrates for microbes may affect microbial ecology and subsequent interaction with the human host. It also raises the question of whether these dietary perturbations may have an effect on subsequent development of some of diseases seen in preterm infants such as NEC, cholestatic liver disease, chronic lung disease or brain white matter disease. Studies in animals suggest that enteral nutrient deprivation leads to a shift in intestinal microbiota to predominantly gram negative Proteobacteria [30]. Along with this is seen an increase in expression of pro-inflammatory cytokines within the intestinal mucosa and loss of the epithelial growth factors which decrease epithelial cell proliferation and increase apoptosis [30]. Highly germane to this study in animals, is recent work done in human infants by different research groups that show a relationship of the Proteobacteria phylum of bacteria in the intestine to the development of NEC in preterm infants [31-33].

### Antibiotics

The use of broad spectrum intravenous antibiotics in very low birth weight infants shortly after birth is very common. In fact, ampicillin and gentamicin are the two most commonly used drugs in neonatal intensive care [34]. Of interest is the fact that recent studies show that the use of antibiotics as well as increased duration of these antibiotics is associated with a greater likelihood of the development of NEC and death [35,36]. The use of antibiotics in these infants has been shown to alter the intestinal microbial ecology [37,38]. The mechanisms of how these alterations may alter host responses are not yet clearly understood, but there are several possibilities. The development of a dysbiosis after prolonged antibiotic treatment may alter the commensal bacteria population found at the surface of the intestine, such that these commensal bacteria no longer provide colonization protection from potential pathogens at the intestinal surface [39-41]. Pathogens can then interact with the intestinal surface including the cells that underlie the epithelial layer that are highly immunoreactive and may result in a brisk inflammatory response associated with inflammation related diseases such as NEC, late onset sepsis as well as the distal organ damage to the liver, lung and the brain.

Although antibiotic stewardship is becoming more common in obstetrics and neonatal intensive care, we still have a long way to go when it comes to overuse of antibiotics. Despite benefits of antenatal antibiotics, these are known to increase late onset neonatal infections [42]. Likewise, the notion that preterm delivery is directly caused by pathogens that may affect the newborn that can also be treated with early antibiotics has never been fully evaluated. The use of early antibiotics in the first days after birth is also correlated with increased prevalence of late onset sepsis by potentially antimicrobial resistant and dangerous microbes such as Pseudomonas, Klebsiella and E. coli [43]. The fact that the microbes can only be found in a small percentage, approximately 2%, [44] in the blood cultures of the babies treated for presumptive sepsis in the immediate newborn, suggests very strongly that there is over use of antibiotics in this population. This is clearly an area that requires further investigation and prompt attention.

### Medications

The human premature neonate has been shown to have low gastric acid output in the first weeks after birth. Gastric acidity is known to be a major non-immune defense mechanism against various infections. Preterm infants have relatively low gastric acid production in the first weeks after birth [45]. Many premature neonates are treated with the class of drugs known as H2 blockers. Mostly these infants suffer from apneic and bradycardic events, which are thought to be related to acid gastroesophageal reflux. Studies have shown that the relationship between gastroesophageal reflux and apnea and bradycardia is actually quite low, approaching approximately 3% [46]. These acid blockers have been shown to increase the odds of development of NEC and have been associated with increased death in these infants [47].

The precise mechanisms of how these affect the intestinal micobiome is not well understood, but recent studies showing that Proteobacteria, the microbes that have been associated with NEC, do not survive well in an acidic environment [48]. Hence, removing this acidic environment may result in a better environment for these bacteria to survive and result in pathogenesis. Along with the data on H2 blockers being associated with greater NEC, there is a small study that showed that acidification of formula resulted in a lower incidence of NEC in preterm infants [49]. Therefore routine use of acid blockers in neonatal intensive care to prevent apneas and bardycardias is not indicated.

### Diet: formula versus human milk

There has been a long term concern about feeding preterm infants with their underdeveloped immune systems human milk that may be colonized with microbes. Commercial formulas are sterilized and do not contain microbes and donor human milk is pasteurized and should contain few if any microbes. Recently, it has become clear that human milk contains up to a wide variety of taxa of microbes, many of which do not originate from the skin of the mother or infant or the infant’s mouth [50-52]. Instead, data suggests that many of these microbes originate from the mothers gastrointestinal tract [52]. This is especially pertinent since manipulation of the maternal microbiota by diet or microbial therapy could be used to promote a breast milk microbial ecology that is most conducive to infant health [20]. Labelled microbes given to pregnant rodents have been found in the mothers’ breast milk and subsequently in the infant [53]. The exact mechanism of transfer to the mothers’ breast remains poorly understood but it has been speculated that the intestinal tract during pregnancy is more permeable than during the non-pregnant state and that microbial translocation may occur readily through this highly permeable intestine. Another mechanism involves the transfer of microbes via dendritic cells which underlie the intestinal mucosa and can send their appendages into the lumen of the intestine and subsequently enter the bloodstream and present these cells to the mothers’ breast [52].

The possibility that these microbes actually play a role in development of the infant intestinal microbiome has been suggested [52] and is highly likely. Of interest is that over time, the microbes from an individual mother’s milk vary only slightly, but the microbes from an individual mother appear to differ markedly from other mothers [50]. This suggests that there may be a milk microbiome that is specific for a certain mother infant dyad and that this specificity could confer benefits to the infant through an enteromammary system, which may offer dynamic immunologic responses from the mother to the newborn [54,55]. These would not be incurred by infants receiving donor milk or formula or perhaps even baby’s own mother’s milk that has been stored in a freezer for prolonged periods. Along the same thinking is the question of whether the technique of “kangaroo care” or “mother skin to skin care” may also offer benefits whereby the mother and infant are exposed to each other’s microbes, and the mother acquires the infant’s microbiota, which may offer specific benefits to the infant immune system.

It is common practice to add fortifiers to either preterm baby’s own mother’s milk or donor milk. The effect of these on the infant intestinal microbiome is poorly understood. Of interest is that there is some literature suggesting that fortifiers derived from human milk rather than bovine milk may be beneficial, [56] the relative effects of these on the developing neonatal intestinal microbiome have not been critically evaluated.

In summary, our concept of the newborn arising from a sterile intrauterine environment and acquiring a microbiome only after it emerges from the birth canal should now be considered somewhat naïve. Large quantities of potentially colonized amniotic fluid with high immunologic and inflammatory potential pass through the fetal intestinal tract. Prenatal exposure to these microbes is likely to have major consequences. These include a disposition to preterm labor, depending on which microbes are present and their functional capability to incite inflammation. Factors that affect this in-utero microbiome such as maternal antibiotics or maternal diet may play a large role in epigenetic and/or immunologic alterations that can occur during this very critical window of development. Perinatal factors such as C-section or vaginal delivery may have long term consequences via differential microbial colonization of the fetus. Various factors in the newborn such as antibiotic or other medication use, diet (breast milk versus formula) or even direct close contact between the mother infant dyad may be of critical importance for future health of the infant. This is an exciting area of current medical science that we are fortunate enough to be a part of.
